# A Cross‐Modal and Cross‐lingual Study of Iconicity in Language: Insights From Deep Learning

**DOI:** 10.1111/cogs.13147

**Published:** 2022-06-04

**Authors:** Andrea Gregor de Varda, Carlo Strapparava

**Affiliations:** ^1^ Department of Psychology University of Milano – Bicocca; ^2^ FBK – Fondazione Bruno Kessler

**Keywords:** Non‐arbitrariness, Phonosymbolism, Iconicity, Cross‐lingualism, Language and vision, Deep learning

## Abstract

The present paper addresses the study of non‐arbitrariness in language within a deep learning framework. We present a set of experiments aimed at assessing the pervasiveness of different forms of non‐arbitrary phonological patterns across a set of typologically distant languages. Different sequence‐processing neural networks are trained in a set of languages to associate the phonetic vectorization of a set of words to their sensory (Experiment 1), semantic (Experiment 2), and word‐class representations (Experiment 3). The models are then tested, without further training, in a set of novel instances in a language belonging to a different language family, and their performance is compared with a randomized baseline. We show that the three cross‐domain mappings can be successfully transferred across languages and language families, suggesting that the phonological structure of the lexicon is pervaded with language‐invariant cues about the words' meaning and their syntactic classes.

## 1 Introduction

A pivotal property of human languages is their ability to refer to entities and events that populate the physical world by means of signs. In oral languages, these signs consist of ordered sequences of sounds; the links between these phonological patterns and the world are determined by both the phonemes that are uttered and their relative position within a word. The nature of the relationships that tie the speech sounds composing a word and that word's meaning has kindled the interest of philosophers since ancient times (see Magnus, 2013, for a historical overview); nonetheless, the deep‐rooted fascination for this puzzling question was waned by the empiricist criticism put forward by Locke (1847) and definitively annihilated by the structuralist axiom of the arbitrariness of the sign (Saussure, 1964). According to the Saussurean perspective on meaning, words should be conceived as arbitrary labels, forced onto the semantic concept they refer to as a result of social processes of cultural transmission. This framework quickly conquered the theoretical panorama (see, for instance, Bloomfield, 1984; Hockett & Hockett, 1960; Levelt, Roelofs, & Meyer, 1999), rejecting a priori the possibility of any natural correspondence between the linguistic sounds and their denotation. However, as noted by Allott (2001), this perspective has restrained the study of the phonological properties of the lexicon beyond the reach of scientific explanation.

The concept of iconic referentiality dismisses the assumption of an arbitrary link between the words and their *denotatum*; it entails that linguistic sounds can bear meaningful associations with their referents, with these associations being mediated not only by the phonological regularities of a given language but also by the sounds' inherent qualities (i.e. their acoustic and articulatory features). Approaches that incorporate iconic principles into lexical semantics are gaining increasing popularity in cognitive research, but they are still conceived as an alternative to the standard view on vocabulary structure. Notably, motivated mappings with the phonological form seem to be rejected a priori only in the lexical domain. However, it is commonly recognized that non‐arbitrary cross‐domain connections account for a variety of linguistic phenomena at different levels of analysis beyond the study of the lexicon. From a syntactic standpoint, it is widely acknowledged that linguistic structures mirror various facets of the structure of experience (Croft, 2002; Haiman, 1985; Levinson, Stephen, & Levinson, 2000). The parallelism between linguistic and temporal sequences has been proposed as an example of this correspondence (Bybee, 1985; Perniss, Thompson, & Vigliocco, 2010): in the sentence “I will eat, shower, and read a book” the hearer will typically infer that the speaker intends to perform the three actions in the order in which they were uttered. Nonetheless, there is no external cue besides the sequential arranging of the verb phrases that support this assumption. Proposals that incorporate iconic principles in linguistic analysis have also been outlined in the domain of morphology, with the observation that for degree adjectives (e.g., *big*, *bigger*, *biggest*) the highest degree of quality is iconically represented by the word with the greatest number of phonemes in its inflection (Wescott, 1971). The claim that a given domain can be structured without any accountable principle is inherently sterile. For this reason, the first research efforts that challenged this view were welcomed with a high resonance in the scientific community.

### 1.1 Phonovisual iconicity

In the late 1920s, the cognitive sciences drew attention to some anecdotal cases that challenged the structuralist principle of the arbitrariness of the sign. Two prominent studies disclosed a non‐trivial link between the participants' guesses about a figure's name and some of its visual properties, namely its shape (Köhler, 1929) or its size (Sapir, 1929). In Sapir's study (1929), participants engaged in a name matching task; they were presented with the images of two tables of different sizes and instructed to pair them with the pseudowords “mil” and “mal.” Intriguingly, the latter phonetic sequence was coupled four times more often with the larger object, showing that the participants' intuitions were biased by the nature of the vocalic phone. In the same year, Köhler showed that the phonological profiles of two non‐words affected their association with two novel shapes: participants tended to label “maluma” a rounded shape and “takete” a spiky one. This latter experiment on shape phonosymbolism had a wide impact on experimental psychology and linguistics: Several studies managed to replicate Köhler's results, corroborating the psychological reality of the so‐called “maluma–takete” effect (Köhler, 1947; Werner, 1948, 2011) or “bouba‐kiki” effect, referring to the pseudowords employed by Ramachandran & Hubbard (2001). These research efforts set the stage for a number of experiments that repeatedly reported the same phonovisual correspondences at different developmental stages (Maurer, Pathman, & Mondloch, 2006; Ozturk, Krehm, & Vouloumanos, 2013; Pejovic & Molnar, 2017) and in various linguistic, geographical, and cultural contexts (Bremner et al., 2013; Ramachandran and Hubbard, 2001; Chen, Huang, Woods, & Spence, 2016; Shinohara & Kawahara, 2010; Ćwiek et al., 2021). The results of the studies on shape and magnitude symbolism were complemented by other findings that related to different properties of the visual modality, such as color (Johanssohn, Anikin, & Aseyev, 2020) and lightness (Hirata, Ukita, & Kita, 2011), to their respective phonetic signs.

Vision is not the only sense by which we experience the world, and several studies have searched for a phonosensory bias in different perceptual modalities. Iconic sensory analogies were then documented in various senses, such as touch (Fryer et al., 2014; Graven & Desebrock, 2018), smell (Atkinson, Speed, Wnuk, & Majid, 2021), kinesthesis (Fontana, 2013), and taste (Gallace, Boschin, & Spence, 2011). Iconic words that make reference to the auditory modality are particularly relevant in linguistic and cognitive research, since their phonosymbolic mapping takes place *within* a modality, relating verbal and non‐verbal sounds. They receive the highest explicit iconicity ratings (Winter et al., 2017), and participants are able to associate them with their meaning with the highest accuracy among all the other sensory modalities (Dingemanse, Reinisch, Schuerman, Tufvesson, & Mitterer, 2016). In the ideophonic lexicon – i.e the portion of the vocabulary that includes marked words depicting sensory imagery (Dingemanse, 2012) – auditory terms are the most prominent class. They occupy the highest rank in the cross‐linguistic implicational hierarchies developed by Blasi, Dingemanse, Lupyan, Christiansen, and Monaghan (2015) and revised by McLean (2021), meaning that if a language does not develop auditory ideophones, it will not produce ideophones related to the other senses. Nonetheless, we chose to focus on vision since we were interested in an analogical iconic mapping that involved a cross‐modal link. With the exception of the auditory modality, the phonovisual biases hold a privileged role among the other senses, both in terms of the research interest they elicited and the consistency of the findings. Indeed, the cross‐modal correspondences in the olfactory‐gustative modality do not seem to be coherent across cultures (Bremner et al., 2013), and the iconic biases in the haptic domain might be mediated by visual imagery (Fryer et al., 2014) and auditory experiences (Winter et al., 2017). In the light of this asymmetry, we approached the multifaceted subject of perceptual iconicity by analyzing the link between phonological profiles and visual features (Experiment 1).

### 1.2 Phonosemantic iconicity

Visual representations do not exhaust the whole semantic spectrum. Several words are grounded in other perceptual modalities, and abstract concepts lack a precise relationship with sensorimotor features in general (Borghi et al., 2017; Crutch & Warrington, 2005; Lupyan & Winter, 2018; Paivio, 2010). Not only are sounds associated with other sensory properties, but they are also more generally associated with lexical meanings. For example, studies have shown that participants are able to couple with an above‐chance accuracy visually presented characters (Koriat & Levy, 1977) and auditorily presented words (Berlin, 1995) of a foreign language with their meaning. Furthermore, it has been shown that participants perform above chance when pairing up words with opposite meanings in languages to which they have not been exposed (Nuckolls, 1999), and when estimating the concreteness of words from languages unknown to them (Reilly, Hung, & Westbury, 2017). Taken together, these findings suggest that the semantic information encoded in a word's phonological profile may include other features that are not exclusively visual. Aiming to extend the scope of our study beyond the domain of perception, we devised a second experiment where we inspected the link between sound and language‐based meaning representations (Experiment 2).

### 1.3 Systematicity

Dingemanse et al. (2015) drew an important distinction between two patterns of non‐arbitrariness in vocabulary structure, namely iconicity and systematicity. The former term reflects the idea that phonemes can convey meaning *per se*, that is, not only through contrastive relations with other sounds but also through their intrinsic sound qualities; in iconic words, aspects of form and meaning are related by means of perceptuomotor analogies. The latter constitutes a different form of non‐arbitrariness prompted by statistical regularities between sound and usage patterns of word classes. Despite its pervasiveness, systematicity has received relatively little attention in linguistics and cognitive science. Systematicity does not concern a direct relationship between phonetic patterns and referential semantic properties; instead, it regards the phonetic regularities that are instantiated within a word class. Class‐level phonetic cues have been found in a broad range of languages, with evidence coming from both typological (Smith, 2011) and corpus studies (Monaghan, Christiansen, & Chater, 2007). Dingemanse et al. (2015) suggested that the phonetic cues that help in discerning between word classes might be language‐specific, featuring ample cross‐linguistic differences. The results from our study (Experiment 3) challenge this assumption, providing empirical evidence that the relationship between phonological profiles and word classes is characterized by significant cross‐linguistic consistency and can be transferred across different language families. Within the computational framework, the analysis of lexical non‐arbitrariness has largely focused on iconicity (Abramova & Fernández, 2016; Abramova, Fernández, & Sangati, 2013; Blasi, Hammarström, Wichmann, Stadler, & Christiansen, 2016; Johanssohn, Anikin, & Aseyev, 2020; Shillcock, Kirby, & McDonald, 2001; Wichmann, Holman, & Brown, 2010; although see Gutiérrez, Levy, & Bergen, 2016; Monaghan et al., 2007; Tamariz, 2008). We believe that the eventual cross‐linguistic consistency of the systematic cues that help in distinguishing between word classes deserves to be addressed at a large scale, and our third experiment aims to fill this research gap.

### 1.4 Relevance

In recent years, non‐arbitrariness has gone from being merely peripheral to the interests of the cognitive science community to being integrated into broader theories of language evolution (Cabrera, 2012; Dingemanse et al., 2013; Ramachandran and Hubbard, 2001), processing (Lockwood & Tuomainen, 2015) and acquisition (Asano et al., 2015; Imai, Kita, Nagumo, & Okada, 2008; Murgiano, Motamedi, & Vigliocco, 2021). A naturally biased relationship between phonetics and semantics restrains the problem space of the evolution of language, positing constraints on the emergence of the vocabulary. Furthermore, a systematic link between a linguistic sound and its referent might strengthen the mnemonic traces in the process of language acquisition (Sathian & Ramachandran, 2019). The effects of phonosemantic correspondences are not encased within language but have been shown to spread to different cognitive faculties, such as categorization (Lupyan & Casasanto, 2015), memory (Ramachandran and Hubbard, 2001), and emotion recognition (Slavova, 2019); moreover, they exert an influence on actional processes such as phonatory behavior (Parise & Pavani, 2011), spatial navigation (Krehm, Maglio, Rabaglia, Seok, & Trope, 2016), and hand grip (Schulman, Vainio, Tiippana, & Vainio, 2013). In the light of their effects within the human cognitive system, the phonosemantic biases are not likely to be limited to a few circumscribed phonetic or semantic clusters, but may instead pervade the lexicon beyond the often reported anecdotal instances.

### 1.5 Aims

In the present study, we tackle the following questions:

- Is there a relationship between the phonological realization of a word and the visual representation of its referent?
- Is this sound‐to‐meaning link extended beyond visual semantics?
- Are word classes organized into consistent phonological clusters?

**Fig. 1:**
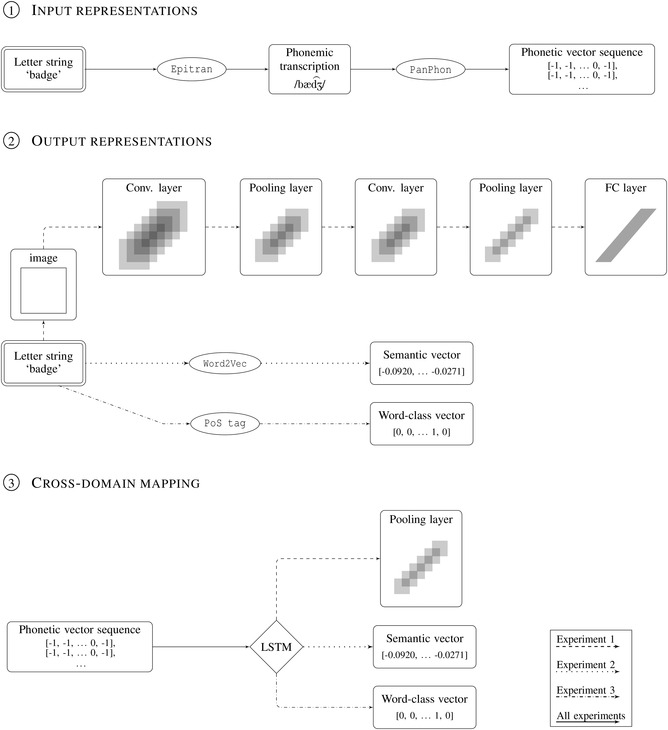
Graphical summary of the experimental pipeline. The sequence in 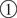 represents the stages of the graphemic‐to‐phonetic conversion, which is common to our three experiments. The flowchart in 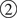 depicts the pre‐processing stages of the images (Experiment 1, upper part of the diagram), the semantic vectorization of the words through Word2Vec (Experiment 2, middle part of the diagram), and the encoding of the word classes (Experiment 3, lower part of the diagram). For typographical reasons, only five layers of the VGG16 network are graphically depicted. The schema in 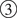 represents the cross‐domain mappings from the phonetic input derived in 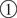 to the output representations obtained in 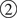. Again, the representations of Experiment 1 occupy the higher position in the diagram, the ones of Experiment 2 are in the middle, and the ones of Experiment 3 are depicted at the bottom (see the legend on the right).

In trying to answer these inquiries, we adhered to three core methodological choices. First, we relied on large‐scale data‐driven procedures, with the intention of assessing the pervasiveness of non‐arbitrariness in a representative linguistic sample, without including human biases in the item selection. Second, we implemented our experiments in a cross‐linguistic setting. We deem that cross‐linguistic diversity is a pivotal testbed for testing the hypothesis of a universal sound‐symbolic substrate underlying all languages, as opposed to language‐specific idiosyncratic systematicity. Third, we configured our experiments as zero‐shot cross‐lingual transfer learning tests, where we trained different long short‐term memory (LSTM)‐based recurrent neural networks in associating phonetic vector sequences with visual (Experiment 1), semantic (Experiment 2), and word‐class (Experiment 3) representations (see Fig. 1). We followed the rationale that if the semantic and syntactic traces contained in the phonetic realization of the lexicon are consistent across languages – hence being a language universal in a broad sense –, then it should be possible to learn a cross‐domain mapping in a set of unrelated languages and transfer it to a novel, typologically distant language without further training.

## 2 Experiment 1

In our first experiment, we explored the idea that vocabulary might be entangled with sensory experience. More precisely, we tested the hypothesis that the phonological properties of the lexicon might be related to the visual world by means of cross‐modal correspondences, and that these correspondences might be consistent across languages. In order to uncover hidden links between these two domains – a task that arguably requires the use of complex transformations –, we trained an LSTM network to associate phonetic vector sequences with visual vectors denoting their referents. The latter were obtained through a forward pass of an image through a pre‐trained hierarchical convolutional neural network (henceforth HCNN; see Section 2.4). The experimental pipeline is summarized in Fig. 1 (dashed line).

### 2.1 Dataset

We performed our first experiment on the THINGS dataset (Hebart et al., 2019), a resource that comprises 26,107 high‐quality naturalistic images depicting a set of 1,854 diverse object concepts. Each item of the dataset was composed of an image paired with a label; these two components were pre‐processed independently, as described in Subsections 2.3 and 2.4. In order to restrict the effects of the morphological noise in the labels, we removed from the dataset all the compound words (305 concrete words, corresponding to 3,839 images).

### 2.2 Translation

Each image label in the resulting dataset was translated into five languages belonging to five language families (see Table 1). We are aware that the choice of translating the labels is not free of concerns: the translation process does not always return the exact same concept in a different language, but rather the concept that overlaps to the highest degree with the original one. However, translating the labels licenses meaningful comparisons across languages – at least, more than employing different language‐specific datasets –, and allows us to align the lexical items across languages, a crucial aspect to take into account when devising disjoint experimental conditions (see Section 2.6). In order to maximize the cross‐lingual coverage of our dataset, while at the same time maintaining a high‐quality translation, we first searched for lexical matches through word2word (Choe, Park, & Kim, 2020), a collection of bilingual lexicons constructed from the publicly available OpenSubtitles2018 dataset (Lison, Tiedemann, & Kouylekov, 2018); then, for the missing items, we employed the ground‐truth bilingual dictionaries based on fastText, released by Facebook Research (Conneau, Lample, Ranzato, Denoyer, & Jégou, 2017). We removed from the analyses the words for which a translation was missing in one or more languages – in other words, we considered only the set intersection of the translated items. The resulting dataset consisted of 16,820 images, depicting a set of 1,161 concrete words. The percentage of translations obtained with each translation tool for all the languages considered in the study is reported in Table 1, along with the percentage of missing items.

**Table 1:** Languages, relative language families, and translation data for each experiment

		Experiment 1			Experiment 2			Experiment 3		
Language	Family	word2word	fastText	Missing	word2word	fastText	Missing	word2word	fastText	Missing
Arabic	Afroasiatic	84.38%	2.71%	12.91%	2.16%	0.41%	97.43%	30.49%	4.55%	64.95%
Hungarian	Uralic	82.18%	3.36%	14.46%	2.01%	0.25%	97.74%	28.87%	3.38%	67.76%
Indonesian	Austronesian	87.42%	1.48%	11.10%	2.12%	0.30%	97.58%	30.23%	3.27%	66.50%
Vietnamese	Austroasiatic	88.26%	0.06%	11.68%	2.12%	0.08%	97.80%	29.89%	0.67%	69.44%
Turkish	Turkic	81.15%	4.65%	14.20%	1.97%	0.36%	97.67%	28.31%	4.17%	67.52%
English	Indoeuropean	NA	NA	NA	NA	NA	NA	NA	NA	NA

### 2.3 Phonetic representations

We derived the phonetic vector sequences corresponding to each image's label through the Epitran‐PanPhon pipeline. In the first step of the procedure, the orthographic text of the label was transliterated into the International Phonetic Alphabet (IPA) with Epitran, a Python package for phonemic transcription. Then, the output was traduced into a sequence of feature vectors with PanPhon, a library that converts IPA segments into subsegmental articulatory features (Mortensen et al., 2016). In line with Jakobson and Waugh (2011), who state that “most objections to the search for the inner significance of speech sounds arose because the latter were not dissected into their ultimate constituents” (p. 182), we chose not to directly hot‐encode the IPA strings. In our opinion, the information‐rich representational format offered by a phonetic feature decomposition is desirable since it uncovers the internal asymmetries that make different phones more or less related to each other (see Blasi et al., 2016; Joo, 2020, for similar considerations). With a hot‐encoding over the IPA vocabulary all the phones would correspond to discrete categories, while two phones might differ by a single feature (e.g., [p] and [b], which are only distinguished by the feature [+/– voiced]), or more than 10 (e.g., [t] and [u], which exhibit 13 different subsegmental features).

The words in the input could be composed of a variable number of phones, which would result in vector sequences of different lengths. To make the tensor shapes comparable, all the input sequences were zero‐padded, with a maximum length of 15. Thus, vector sequences derived from words with less than 15 phones were extended with zeroes, which would be subsequently hidden in the masking layer of the LSTM network (see Section 2.5). On the other hand, vector sequences corresponding to words with more than 15 phones were truncated, and the phonetic vectors corresponding to the following phones were discarded. Note that the items with 15 phones or more were less than 0.03% of the total, so the number of truncated words was negligible.

### 2.4 Visual representations

To transform the raw RGB images in the input into cognitively inspired visual representations, we relied on VGG16, an HCNN for large‐scale image recognition (Simonyan & Zisserman, 2015). HCNNs exploit the hierarchical nature of the visual data to assemble representations of increasing complexity using small and simple patterns repeated across the images in input. They are biologically inspired models (LeCun & Bengio, 1995; LeCun et al., 2015) that have been developed in the field of computer vision with the purpose of classifying images, predicting a label from the pixel‐wise RGB codes in input (Krizhevsky, Sutskever, & Hinton, 2012). HCNNs are usually composed of stacked convolutional and pooling layers, followed by standard fully connected (FC) layers (Simonyan & Zisserman, 2015). Convolutional layers create feature maps that represent in a distributed tensor format the presence of features of various levels of abstraction in the input; these features are extracted through the application of learned filters to input images. Pooling layers then serve the purpose of lowering the resolution of the feature maps. Since the absolute position of a certain feature forming a motif might vary, coarse‐graining each feature's position can create invariance to small shifts and distortions (LeCun et al., 2015). In deep models, shallow layers usually learn low‐level visual features (e.g., lines, edges, color blobs) while layers that are deeply embedded in the network can extract high‐level attributes (e.g., object parts, textures). The final layers then encode images as complex representations (e.g., object shapes) which are employed for the ultimate purpose of the network, that is, classification (Mahendran & Vedaldi, 2015).

The visual vectors included in this study consisted of the outputs of the fifth max‐pooling layer of the pre‐trained network, in response to a forward pass of each image in the dataset. After freezing all the model's weights by setting it in evaluation mode, we fed each image x in our stimulus set through the VGG16, in order to extract the resulting feature maps φ(x) of block5_pool; the resulting vectors were in turn flattened before being processed by the LSTM model. The weights of VGG16 were configured according to its pre‐training on ImageNet (Deng et al., 2009). We employed the output of the VGG16 network as an approximation of a representational format proper to the human perceptual system. Indeed, HCNN‐based representations can be successfully mapped onto neural responses to visual stimuli at different levels of processing within the ventral stream, even if the networks are not explicitly optimized to fit neural data (Yamins & DiCarlo, 2016). From a psychological perspective, these representations have been proposed to be cognitively plausible at least at the computational level of description (Marr, 1982), being able to predict human behavior and performance in several tasks (Günther, Petilli, Vergallito, & Marelli, 2020; Günther, Marelli, Tureski, & Petilli, 2021; Günther, Petilli, Vergallito, Ciapparelli, & Marelli, 2021).

### 2.5 Neural architecture

An LSTM was trained to associate the sequences of phonetic feature vectors in input into the visual vectors in output, with a many‐to‐one topological structure. The choice of the architecture was motivated by the nature of the input that LSTMs can process: while standard feedforward neural networks can only treat single data points, LSTMs are endowed with feedback connections, that enable them to process inherently sequential data – in our case, the chains of phonetic vectors. The model was configured with Keras, a deep learning framework for Python (Chollet & others, 2015); it comprised a masking layer, followed by a single LSTM layer with 500 neural units, a dropout of 0.2, and a recurrent dropout of 0.2. The LSTM layer was connected to a dense layer with the number of units (25,088) matching the dimensionality of the target visual vector and equipped with rectification non‐linearity (Rectified Linear Unit, *ReLU*). Cosine similarity was employed as both the objective function and metric, and the Adam optimization method was employed for training (Kingma & Ba, 2014), with the learning rate set to 0.01. All the hyperparameters described above were set without tuning. Random seeds were set for replicability purposes.

### 2.6 Experimental conditions

**Fig. 2:**
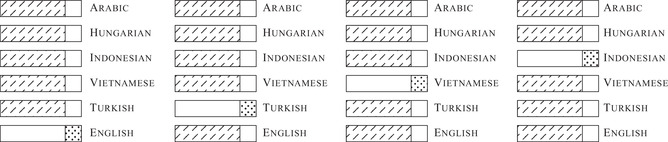
Schematic summary of the train‐test combinations in our experimental conditions. For typographical reasons, only four out of six conditions are reported. The rectangular shapes represent the totality of the word‐image pairs available in each language. The training sets are marked with north‐east lines, whereas the test sets are represented with the dotted patterns. As depicted in the figure, the training and test sets are completely disjointed. In the first block on the left, the training set is composed of a subset of the Arabic, Hungarian, Indonesian, Vietnamese, and Turkish data, whereas the test set is a different subset of English data.

We structured the experimental conditions following two main principles aimed at limiting the effects of the etymological relatedness of the items in the training and the test sets as much as possible. First, we prevented an image and a concept to occur in both sets, by randomly splitting the labels into two subsets, with a train‐test split ratio of 0.8. Following this partition, the training set consisted of 929 concepts depicted by 13,397 images, whereas the test set was composed of 232 concepts represented by 3,423 images. Then, we devised our conditions so that the language on which the network was tested did not overlap with the set of languages on which the training was performed. In other words, in each condition the model was trained in the concatenation of the training sets of five languages ${\left\{L_{i}\right\}}_{i=1\text{…}5}$ and tested in the test set of a sixth language *L_6_* which was excluded from the training set, following a non‐random sixfold cross‐validation procedure (see Fig. 2). Each model was trained for one epoch in the five languages in the training set to map the phonetic vectorization of a word denoting an image to the convolution‐based transformation of that image; then, it was tested on the same task in a novel language, belonging to a different language family. The experimental conditions were thus constructed so that the training and test sets were disjoint with respect to the concepts, the images representing them, and the languages to which the labels were translated. The experimental models' performances were assessed by comparing their results with the ones obtained by a parallel random model, which defined a baseline for quantifying the increase in performance due to the relevant multilingual signal. Concretely, the parallel model was trained on a dataset where the correspondence between input and output vectors was randomized by shuffling the visual vectors in output. All models were trained on 66,985 samples (13,397 × 5 languages) and tested on 3,423 items.

### 2.7 Results

The results of the six cross‐lingual models and their random counterparts are reported in Table 2. The descriptive statistics in the first columns reveal that across all the experimental conditions, the cross‐lingual models always outperformed their randomized baselines. To test whether this pattern of results was associated with statistical significance, we contrasted the results of the random and the cross‐lingual models through a set of paired samples *t*‐tests between the element‐wise cosine similarity of the target visual vector in output with the predictions of the two alternative models, for each experimental condition. The inferential tests confirmed the soundness of the descriptive results, with all the contrasts being statistically significant. Furthermore, across all the experimental conditions, the 95% confidence intervals (CI) of the cross‐lingual models did not overlap with the corresponding CI of the parallel random models. This suggests that the sound‐to‐vision correspondences inherent in the phonological structure of the lexicon can be learned in any direction and generalized to all the languages included in our study. All the contrasts were associated with medium‐to‐large effect sizes, with the exception of the zero‐shot transfer to the Vietnamese language.

**Table 2:** Results by experimental condition (Experiment 1)

	Cross‐lingual Model			Randomized Baseline			Contrast		
Language	Cosine	*SD*	95% CI	Cosine	*SD*	95% CI	*t*	*p*	*d*
Arabic	0.2343	0.0411	[0.2329,0.2357]	0.2243	0.0382	[0.2230,0.2256]	10.42	≪.0001	0.6600
Hungarian	0.2382	0.0410	[0.2368,0.2396]	0.2278	0.0386	[0.2266,0.2291]	10.78	≪.0001	0.6321
Indonesian	0.2391	0.0394	[0.2378,0.2404]	0.2243	0.0399	[0.2229,0.2256]	15.45	≪.0001	1.4385
Vietnamese	0.2320	0.0431	[0.2306,0.2335]	0.2224	0.0384	[0.2211,0.2237]	9.77	≪.0001	0.4544
Turkish	0.2381	0.0404	[0.2367,0.2394]	0.2257	0.0387	[0.2244,0.2270]	12.99	≪.0001	1.1956
English	0.2389	0.0418	[0.2375,0.2403]	0.2228	0.0384	[0.2216,0.2241]	16.60	≪.0001	1.3220

*Note*. The first column of the table specifies the language of the fold on which the validation was performed, implying that the training had been carried out on all the languages but the one in the test set. The following six columns of the table present the mean, the standard deviation (*SD*), and the 95% confidence intervals (CI) of the cosine similarity between the target visual vector and the cross‐lingual or the random model's prediction for every item in the test set. The last three columns indicate the statistics of the contrast between the two models' results (*t* statistic, *p*‐value, and Cohen's *d*).

### 2.8 Discussion

Our LSTM imaginative models, trained on multilingual data to induce a mapping between phonological profiles and sensory representations, showed the ability to learn cross‐modal and cross‐linguistic correspondences in the lexicon, suggesting that visual information is implicitly encoded in the phonological structure of linguistic data. Showing a link between *meaningful* speech sounds and visual representations, we complement the behavioral studies presented in the Introduction, which disclosed a powerful and consistent link between *meaningless* speech sounds and magnitude, color, and geometrical shape. Across our experimental conditions, the LSTM networks were able to engage in a generative process where their visual imagery reproduced real‐world concrete representations better than what would be expected by chance. Our strict manipulation of the linguistic distance between the languages in the training and the test set allows us to rule out the effect of any etymological relatedness between the different languages' vocabularies.

## 3 Experiment 2

In our first experiment, we showed that the phonological structure of the concrete lexicon is entangled with a visual experience. Nonetheless, concrete and vision‐related words constitute only a fraction of the whole vocabulary; additionally, even the words that refer to visually perceivable objects are not completely summarized by their visual attributes. For instance, the conceptual representation of the word *cloud* is not fully specified by a mental picture of a puffy white shape. We have auxiliary knowledge about their nature – for instance, the fact that they are mainly composed of water particles suspended in the atmosphere – we have acquired through language use and that is part and parcel of the word meaning. Aiming to extend our previous study beyond the perceptual domain, we trained an LSTM model to associate the phonetic with the corresponding language‐based semantic representation of a word, encoded as a 100‐dimensional word embedding. Word embeddings represent word meanings as high‐dimensional vectors, usually extracted from large corpora of natural language data. These representations are rooted in the theoretical foundations of the distributional hypothesis, according to which the semantic similarity between words is a function of the similarity between the contexts in which they occur (Firth, 1957; Harris, 1954). Word embeddings do not consist of a coarse representation of the context in which the word occurs, but rather in an abstract structure that accumulates from encounters with lexical items and their context (Lenci, 2018). Different from visual vectors, they can be computed for every word in a corpus, and thus are suitable for extending our inquiry beyond the scope of visual semantics. The experimental pipeline of our second experiment is depicted in Fig. 1 (dotted line). The phonetic vectorization of the graphemic sequences in the input was identical in every aspect to our previous experiment. Hence, we redirect the reader to Section 2.3 for a detailed description of the procedure.

### 3.1 Semantic representations

The semantic representations employed in this experiment consisted of pre‐trained word embeddings generated with word2vec (Chen, Mikolov, Corrado, & Dean, 2013) from the British National Corpus, and released by Rei and Briscoe (2014). The representation learning tool was based on the skip‐gram model, which inputs a sequence of words into a log‐linear classifier with a continuous projection layer, trained to predict words within a window size of five. Semantic vectors were available for 1.93M words.

### 3.2 Translation

The words for which a semantic vector was available were translated following the same pipeline as in Experiment 1. In this case, the procedure resulted in a severe data loss (see Table 1); nonetheless, the original dataset was much larger with respect to the THINGS database, and although it was not possible to obtain a translation for the vast majority of the items, the set intersection of the translations comprised 24,612 words.

### 3.3 Neural architecture

In the present experiment, in the light of the reduced dimensionality of the semantic with respect to the visual vectors, we constructed a model with an LSTM layer comprising 50 hidden units. The LSTM layer was followed by a dense layer with an equivalent shape. All the other hyperparameters were left unaltered with respect to the previous experiment.

### 3.4 Experimental conditions

We organized our experimental conditions following the same procedure as in Experiment 1. We divided the 24,612 concepts into a training (19,690 items) and a test set (4,922 items), with a 0.8 train‐test split ratio. Then, we constructed six conditions where we trained the LSTM networks in the concatenation of the training set data in five languages and tested it in the test set relative to the language that was excluded from training. Once again, the models were tested in a data sample where the concepts, the semantic vectors, and the language were not represented in the training set. The results of the experimental models were compared with the ones achieved by a randomized baseline, trained on datasets where the correspondence between the phonetic vectors in input and the semantic vectors in output had been shuffled. All the models were trained on 98,450 samples and tested on 4,922 items.

### 3.5 Results

Table 3 reports the results of the models paired with their random counterparts. With the only exception of the transfer to Vietnamese, all the models outperformed their randomized baselines, with the contrast between the two models reaching statistical significance (although with marginal effect sizes). The above‐chance performance of the cross‐family networks is consistent with the hypothesis that a certain amount of cross‐linguistic correspondence between form and meaning is stable across languages, and thus can be exploited when predicting a word's meaning in a previously unseen language. Moreover, this correspondence is not limited to visual vocabulary (Experiment 1) nor a subset of culture‐independent concepts (Blasi et al., 2016; Wichmann et al., 2010) but can be captured at a lexicon‐wide level. With respect to our previous experiment, the present results show that phonological patterns also reflect a component of meaning that is encoded in language use, as reflected by a distributional semantic representation.

**Table 3:** Results by experimental condition (Experiment 2)

	Cross‐lingual Model			Randomized Baseline			Contrast		
Language	Cosine	*SD*	95% CI	Cosine	*SD*	95% CI	*t*	*p*	*d*
Arabic	0.5263	0.0687	[0.5244,0.5282]	0.5245	0.0654	[0.5227,0.5263]	3.6849	.0002	0.0525
Hungarian	0.5253	0.0683	[0.5234,0.5272]	0.5241	0.0665	[0.5222,0.5259]	4.1277	≪.0001	0.0588
Indonesian	0.5264	0.0690	[0.5245,0.5284]	0.5237	0.0675	[0.5218,0.5256]	9.4220	≪.0001	0.1343
Vietnamese	0.5214	0.0742	[0.5193,0.5234]	0.5218	0.0705	[0.5199,0.5238]	−1.2914	.1966	−0.0184
Turkish	0.5256	0.0675	[0.5237,0.5275]	0.5230	0.0693	[0.5210,0.5249]	7.7257	≪.0001	0.1101
English	0.5281	0.0683	[0.5262,0.5300]	0.5227	0.0701	[0.5207,0.5246]	15.1285	≪.0001	0.2156

## 4 Experiment 3

In our previous experiments, we showed that our LSTM models were able to detect iconic cues in the input and exploit them when making predictions about the physical‐geometrical properties of a word's referent and its language‐based semantic representation. In our third experiment, we aimed to investigate whether the sound of a word encoded consistent cues about its syntactic behavior, expressed as its word class. It is widely accepted that word classes share relevant phonological properties *within* languages; nonetheless, the dominant view on systematicity in vocabulary holds that these properties fluctuate *across* languages (Dingemanse et al., 2015). We wanted to test the hypothesis that word classes might instead be organized according to consistent phonological principles, with some structural limitations on their cross‐lingual variation. To address this issue, we relied on transfer learning in a classification setting, training an LSTM model to associate phonetic vector sequences with the hot‐encoding of their word classes.

Since we did not modify the procedure for obtaining phonetic vectors from the letter strings in input, a description of the methodology can be found in Subsection 2.3. The experimental pipeline is depicted in Fig. 1 (dashed‐dotted line).

### 4.1 Word‐class representations

The word‐class representations employed in the present experiment consisted of the one‐hot encoding of the part‐of‐speech (PoS) tags of the vocabulary of the British National Corpus, released by Kilgarriff (1997). We collapsed the original 54 tags into 11 coarse supertags, which corresponded to the dimensions of the hot‐encoded embedding. From the original database (208,656 items), we removed all the words with an ambiguous PoS tag (i.e., that were associated with more than one syntactic label); the resulting list comprised 152,855 items.

### 4.2 Translation

The words associated with a univocal PoS tag were translated into five languages with the combination of word2word and fastText, following the same procedure as in our previous experiments. The number of items for which a translation was available in all five languages was 24,246. No infinitive marker survived the translation procedure, so we removed the corresponding dimension from the vectors. With this procedure, we assumed an alignment of word classes across languages. This is a rather strong assumption, which is not likely to be fully supported by our data; however, different language‐specific PoS taggers often classify words according to different tag sets, whereas our experimental procedure called for a shared classification schema.

### 4.3 Neural architecture

Given that the experimental setting consists of a multiclass classification problem, categorical cross‐entropy was used as the objective function, and for the same reason the softmax activation function was adopted for the output layer, and accuracy, precision, and F1 score were employed as metrics. After the removal of the infinitive markers, the vectors in the output were 10‐dimensional arrays; thus, the output layer was equipped with 10 neurons. In the light of the reduced dimensionality of the word‐class vectors in the output, we reduced the size of the LSTM layer to 25 units. All the remaining hyperparameters were left unaltered with respect to our previous experiments.

### 4.4 Experimental conditions

In order to construct our experimental conditions, we first split the 24,246 original items in a training set and a test set, which both included 12,123 instances. We employed a 0.5 split ratio in this experiment in order to have a reasonable number of instances in the test set for the minority classes: while upsampling is a useful procedure for balancing the classes in the training set, there is no use in upsampling the items in the test set. Thus, we dealt with class imbalance by randomly oversampling all the classes but the majority one in the training set, which reached 64,600 items. The usual six experimental conditions were devised by concatenating the training data in all the languages but one, and employing as test set the test data in the language that had been excluded from the training. The results of the experimental models were assessed by comparing their performances to the ones obtained by the parallel randomized baselines, where the order of the word‐class vectors in output had been shuffled. All the models were trained on 323,000 instances and tested on 12,123 samples.

## 5 Results

Table 4 reports the results of the transfer of the LSTM‐based classifiers. The first three columns indicate the accuracy, the weighted precision, and the weighted F1 score obtained by the cross‐lingual models, whereas the following three columns specify the same performance indexes relative to the randomized baseline models. The significance of the contrast between the accuracy of the parallel models was assessed by means of the McNemar test, a statistical test employed on paired nominal data. In all cases, the standard $\chi^{2}$ calculation was employed, since the number of observations did not require us to resort to the exact binomial test. In all the experimental conditions, the cross‐lingual models outperformed the randomized baselines by a wide margin in all the metrics considered, with all the contrasts reaching statistical significance.

**Table 4:** Results of the transfer of the neural classifier

	Cross‐lingual Model			Randomized Baseline			Contrast	
Language	Accuracy	Precision	F1	Accuracy	Precision	F1	$\chi^{2}$	*p*
Arabic	0.1001	0.4194	0.1396	0.0155	0.0009	0.0017	794.7239	≪.0001
Hungarian	0.1462	0.4018	0.1828	0.0168	0.0008	0.0014	1278.9005	≪.0001
Indonesian	0.1208	0.4419	0.1717	0.0183	0.2804	0.0049	971.0473	≪.0001
Vietnamese	0.0880	0.4060	0.1311	0.0159	0.0009	0.0017	622.6544	≪.0001
Turkish	0.1933	0.4481	0.2570	0.0286	0.3316	0.0056	1637.8704	≪.0001
English	0.1127	0.3977	0.1498	0.0360	0.3911	0.0233	503.9111	≪.0001

*Note*. We do not report recall scores since weighted recall is mathematically equivalent to accuracy in multiclass classification.

### 5.1 Discussion

The results presented in the previous section are in line with our predictions and provide empirical evidence in favor of the existence of a universal phonetic substrate underlying word class distinctions across languages. To our knowledge, the present study constitutes the first attempt to refute the idea of a within‐language idiosyncrasy in lexical systematicity through computational methodologies and at a large scale. We showed that the relationship between phonological profiles and word classes can be effectively transferred across language families, yielding language‐independent generalizations in the mapping. Hence, systematicity should be regarded as a candidate universal feature underlying word formation. The view that iconic links are shared across languages should then be complemented by the finding that the phonological profiles of the lexical items are linked not only to their meaning but also to their organization in grammatical and distributional clusters.

### 5.2 Follow‐up analyses

**Fig. 3:**
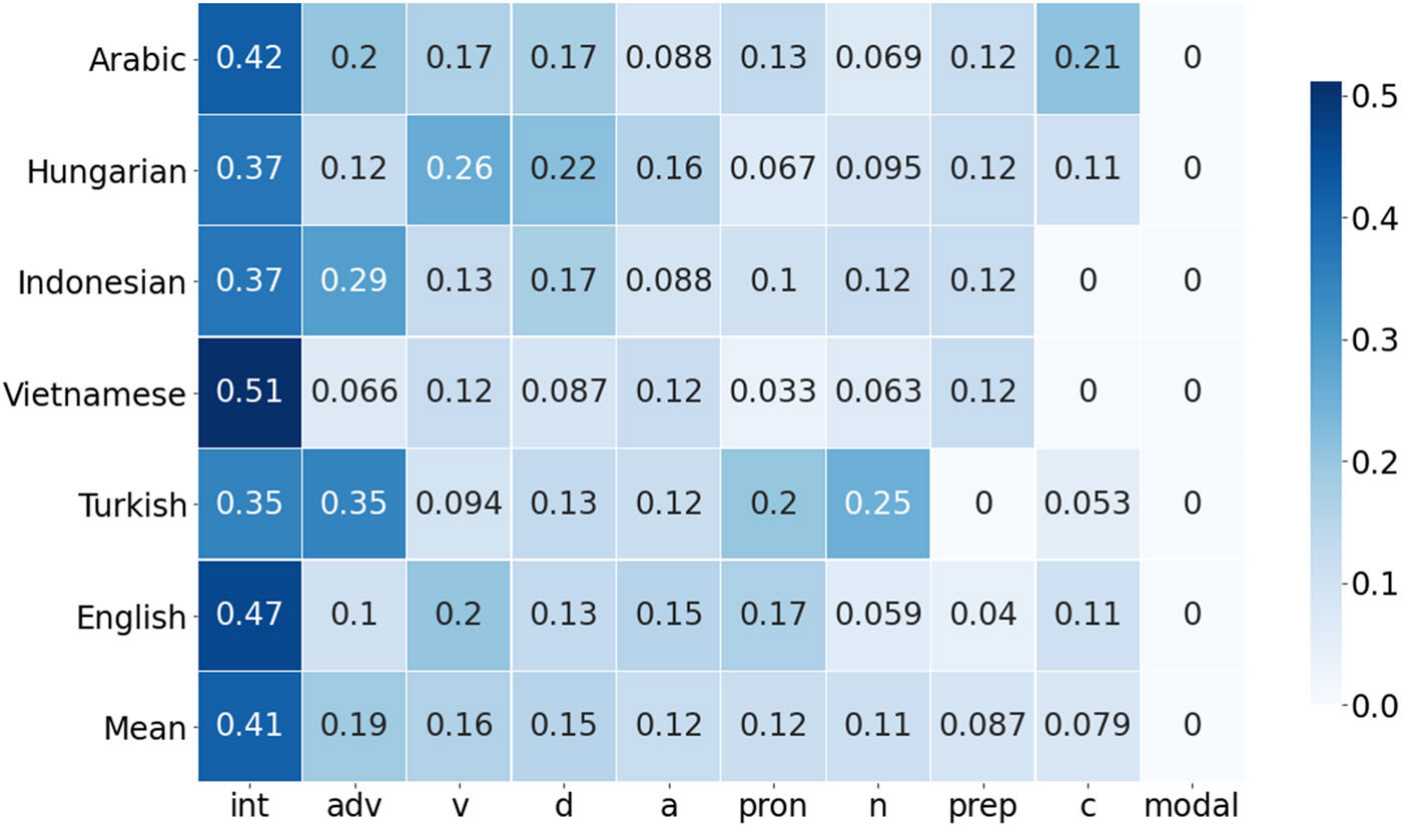
Transfer accuracy averaged by PoS tags.

Once we verified that word classes are characterized by cross‐linguistically stable phonological clusters, a natural question that arises is whether phonosyntactic information is uniformly distributed across syntactic categories, or whether some grammatical clusters incorporate stronger correspondences with their phonetic realization. To do so, we calculated the average accuracy of the cross‐lingual models for each PoS in our tagset. The results are summarized in Fig. 3, which reports the accuracy aggregated by the PoS tag for each of the languages, as well as a second‐order mean across all six languages considered in the study. Overall, our results are consistent across languages: the average pairwise correlation between the PoS‐aggregated accuracy in all the combinations of two languages is *r* = .7738. The word class predicted with the highest accuracy by the cross‐lingual models are interjections. This result is not surprising: Interjections directly express instinctive reactions (Bloomfield, 1984) and can be closely related to their spontaneous manifestation (Wharton, 2003); hence, it is natural to find a more transparent link between their phonoarticulatory expression and their class. Furthermore, this result is aligned with various findings documented in the literature. For instance, interjections are explicitly judged as the most iconic PoS by English speakers (Winter et al., 2017), and the interjection “Huh?” shows a particularly stable phonological realization, being found in roughly the same form in spoken languages across the world (Dingemanse et al., 2013). The accuracy in the other PoS does not seem to follow any clear pattern with respect to the linguistically relevant distinction between content and function words. For instance, adverbs are predicted with very high accuracy, but they can behave as both function and content words. Then, verbs, which are mostly content words with the exception of auxiliaries, occupy the third position in the scale, but are immediately followed by determiners. This suggests that the phonosyntactic clustering encoded in the phonological structure of the lexicon is subjected to a more subtle distinction than the coarse contrast between function and content words. The accuracy averaged by PoS that we obtained in the classification task is coherent with the cross‐linguistic kinship of ideophones to other syntactic classes (Dingemanse, 2021). Ideophones are often connected to – or realized as – adverbs (for instance in Gbaya, as reported by Roulon‐Doko, 2001), verbs (as in Shona, see Fortune, 1971), and adjectives (as in Ewe, see Ameka, 2001). These syntactic classes obtained comparably high‐performance scores in our analysis, occupying the second, third, and fifth positions in our ranking. The high‐performance scores obtained for determiners are reminiscent of the well‐known role of iconicity in deictic demonstratives (Johansson & Zlatev, 2013; Johansson & Carling, 2015), where the pitch is associated with spatial distance (Ultan, 1978; Traunmüller, 1994; Woodworth, 1991). However, an important distinction must be made with respect to the correspondence between our findings and the ones we just reported. In Experiment 3, we showed that the words' sounds can be associated with their syntactic class, with this association varying in strength across word classes. The studies we summarized above showed instead that word sounds have a different association with their meaning as a function of their syntactic class. These two kinds of relationships are inherently different: The instances of a syntactic class can vary a lot in their meaning, and this is particularly clear for open lexical classes. Nonetheless, we showed that some word classes display a certain level of cross‐linguistic phonetic regularity, and the same classes have been shown to exhibit a high internal consistency in the mapping between sound and meaning. Taken together, our results and the findings presented in previous literature show an interesting convergence of systematic and iconic information, where syntactic classes with a more distinctive phonetic profile are also characterized by higher phonosemantic transparency. Our last study thus complements the body of findings demonstrating that iconic phonological patterns are not uniformly distributed in the lexicon, and shows that this asymmetry is mirrored by the way in which different languages encode grammatical classes through phonology.

## 6 Conclusion

This research effort constitutes a large‐scale deep learning‐based analysis of non‐arbitrariness in language. Our key contribution consists in showing that the vocabulary is structured in partial resemblance to the visual world and contains phonological cues about the meaning of a word and its syntactic class.

Iconicity implies a meaningful link between form and meaning, such that the relationship between the two domains can be described as analogical. Unfortunately, our experimental approach does not ensure that the relationships learned by these models are analogical in nature. In fact, the general opaqueness of the deep learning methods we employed prevents us from understanding the kind of correspondences that have been exploited by the networks in order to make their predictions. For some of the anecdotal studies that we reported in the Introduction, the authors were able to interpret the link between the sound and meaning they detected. For instance, in the bouba‐kiki experiment, the round vowel /u/ might suggest the presence of rounded shapes in its referent, in a quasi‐synesthetic analogical relationship. Unfortunately, scaling up to nearly the whole lexica of six different languages makes the explanation of these correspondences an unrealistic enterprise, and the phonovisual, phonosemantic, and phonosyntactic biases that our networks exploited in their predictive behavior elude a precise explanation. However, it should be noted that the cross‐lingual setup of our experiments mitigates the problem. If our experiments were performed within a single language, the finding that similar sounds express similar meanings could not be directly ascribed to iconicity, as it could be simply driven by the affixation of certain bound morphemes to the same roots. For instance, the word *clearly* is both semantically and phonologically close to the word *clear*, but this relationship is not dependent on sensorimotor analogies, but simply on the fact that the two words share the same root. On the other hand, if our experiments were performed on different languages belonging to the same language family, another possible source of non‐arbitrariness that is not iconic in nature could be the etymological relatedness between words in typologically close languages. However, there are not many reasons that can account for a large‐scale correspondence between sound and meaning that is detected across language families. First, this correspondence could be iconic in nature, as we have described it insofar. While the finding that iconic links are shared across different language families might be surprising at first, their cross‐lingual consistency is nonetheless predicted by their own definition. Iconic biases are related to sound and meaning representations by means of perceptuomotor analogies (Dingemanse et al., 2015). Being grounded in the sensory and motor systems, which are relatively culture‐invariant components of human cognition, there are no reasons to expect them to display an ample degree of variation across languages. Closely related to the iconic account for the form‐meaning association biases is the indexical explanation. Words can be related to their associated sensory experience by means of direct resemblance but also on account of their co‐occurrence (Dingemanse, 2021). For instance, when looking at a perceptually pleasing landscape, one may utter the vocalization “wow.” This phonetic sequence does not bear any resemblance with the visual features of the landscape but is a typical *response* to an enchanting view, which routinely follows it. Cases of this kind might constitute a portion of the items in our second and third experiments (especially in the case of interjections), but we doubt they could have played a role in our first study. The image labels in the input were not typical responses to the presentation of those images, but their names; hence, this semiotic alternative is not likely to have been a major determinant of our results. Alternatively, the sound‐to‐meaning link could relate to some functional constraints in the interactional environments in which speakers of different languages communicate. Similar communicative environments might lead to the independent evolution of similar ways of referring to things, as similar physical environments lead to the development of similar body plans (see Dingemanse et al., 2013). A third possibility holds that similar words would display similar phonological patterns for being underpinned by a common genetic infrastructure. This view has been proposed within the literature on interjections (Müller, 1873; Sapir, 1921; although see Dingemanse et al., 2013) but is difficult to support at a lexicon‐wide level: positing innateness for a wide variety of linguistic items would hardly be realistic given the timescale involved in language evolution (Dingemanse et al., 2013; Thompson, Smith, & Kirby, 2012). Hence, we believe that the only reasonable alternatives that can account for our findings are the interactional and iconic explanations. The present work is not sufficient to disentangle these hypotheses, and we leave to future research an empirical test of their predictions.

The difficulty in interpreting the nature of the correspondences learned by our models is not confined to our first two experiments on phonovisual and phonosemantic iconicity. The cross‐lingual stability of the word‐class cues that characterize terms with similar syntactic behavior challenges the assumption that systematicity should be regarded as an idiosyncratic linguistic feature; however, the reason for this phonological coherence within word classes is not unequivocal. Indeed, we can identify in this condition the same theoretical alternatives that we highlighted above. We speculate that this finding might point to the role of sensorimotor processing in shallow syntax. For the word‐class cues to be relatively invariant across languages, they must be rooted in (or at least related to) other domains of the human cognitive system that show a certain degree of cross‐cultural stability, and the perceptual and the motor systems are ideal candidates with that respect. Alternatively, a more functional approach would posit for word classes usually uttered in similar interactional contexts to display a certain degree of similarity in their phonological realization. For instance, we could expect word classes employed more often or learned earlier during language acquisition to display phonemes that are easier to produce and process. Again, a contrast between these two alternatives falls beyond the scope and the explanatory power of this study, and we leave their proper assessment to future research.

Taken together, our findings show that a remarkable amount of information is encoded in a word's sound: phonological profiles seem to contain cues concerning not only its meaning but also its syntactic behavior. A phonetic representation should not be seen as a formal and symbolic transposition of a word's meaning, but rather as an iconic pointer to its perceptual, semantic, and syntactic representation. While the present work highlighted the pervasiveness of linguistic iconicity, the decoupling of form and meaning must be recognized as a fundamental feature of language as well: without a certain degree of arbitrariness, it would not be possible to denote a potentially infinite set of concepts and their relationships (Lockwood & Dingemanse, 2015). Arbitrary and iconic principles should be regarded as distinct properties of language (Sidhu & Pexman, 2018), with the complementary functions of detaching and grounding it to the sensorimotor experience.

## Conflict of interest

The authors declare no conflicts of interest.
